# COVID-19: studying the global pandemic – foreword

**DOI:** 10.2217/fvl-2020-0211

**Published:** 2020-07-27

**Authors:** Stefano Rusconi, Frederick G Hayden

**Affiliations:** 1 ^1^Associate Professor in Infectious Diseases, DIBIC Luigi Sacco, University of Milan, Milano 20157, Italy; 2 ^2^Stuart S. Richardson Professor Emeritus of Clinical Virology & Professor Emeritus of Medicine, University of Virginia School of Medicine, Charlottesville, VA 22908, USA

**Keywords:** asymptomatic infection, coronaviruses, COVID-19, cytokines, evolution, inflammation, public health, transmission

This special issue of *Future Virology* contains nine articles on diverse aspects of the COVID-19 pandemic and its causative agent, SARS-CoV-2. The topics range from basic virology on coronavirus evolution and replication to identification of repurposed therapeutics for clinical testing to public health issues including the conundrums of asymptomatic viral transmission and risks to homeless populations. While several of the reports contain original data, others are Reviews, Special Reports or Commentaries. Given the response of the global research community to the pandemic, we have received unprecedented submissions in this area and have therefore compiled these into one issue to provide a useful resource to those working in the field. While readers should be aware that all but one of the articles have undergone external peer review, as per the journal’s usual policies, we should point out that the conclusions and opinions of some of the authors are speculative.

The Commentary by Parvez [1] briefly reviews the detection of SARS-CoV-2 RNA in fecal samples, including its persistence, and the finding of gastrointestinal complaints in a minority of hospitalized patients. Others have also highlighted that some COVID-19 patients present primarily or exclusively with gastrointestinal manifestations. Given the presence of ACE-2 receptors in intestinal epithelial cells and the documentation of SARS-CoV-1 infection involving the gastrointestinal tract, it is not surprising that the SARS-CoV-2 might also cause such infections and associated illness. Of course, fecal aerosols were implicated in a very large SARS outbreaks in Hong Kong in 2003. However, infectious SARS-CoV-2 has been only rarely isolated from fecal samples to date, and the possible risks of fecal–oral or fecal–aerosol transmission remain to be clarified. Interestingly, Parvez points out the greater expression of ACE-2 receptor in cholangiocytes than hepatocytes in COVID-19 patients and speculates about how this and other mechanisms, particularly immune-mediated ones, might be contributing to the substantial frequency of hepatic abnormalities seen in hospitalized patients. Of course, other factors including hypoxemia, ischemia and medication side effects are also likely to be contributory. The latter concern has led to premature discontinuation of intravenous remdesivir therapy in hospitalized COVID-19 patients. The immunopathologic effects of pro-inflammatory cytokines like IL-6 are the subject of ongoing randomized, controlled trials in severe COVID-19.

The thoughtful Commentary article by Conway *et al.* [2] addresses the extraordinary challenges of mitigating COVID-19 risks in homeless persons in central Vancouver (BC, CA, USA). Like many other inner city populations, the one in Vancouver has high frequencies of psychiatric disorders, opiate drug dependence and overdose-related deaths. Sadly, the latter increased temporally with loss of many supportive services in efforts at social distancing, including loss of daily directly observed drug administration. The authors describe the difficult choices faced by city administrators and private entities in balancing infection risks to staff and maintenance of essential services. They then propose specific strategies to improve current circumstances and also emphasize the long-term need for stable housing. These recommendations are highly relevant for essentially all urban centers that harbor such populations of high-risk persons and reflect more broadly the challenges in mitigating infection risks in other disenfranchised populations, like those in refugee camps. Of course, once a safe and effective SARS-CoV-2 vaccine becomes available, immunization of such high-risk populations will be a priority, as has been shown by the deployment of hepatitis A virus vaccine to mitigate outbreaks in homeless populations in North America.

Yang *et al.* [3] report the case of a 57-year-old patient who had a positive RT-PCR for SARS-CoV-2 after three negative sputum tests in the context of mild illness. The patient was caring for her father from 1 to 6 February 2020, after her mother died from cardiac complications. Her father was diagnosed with COVID-19 on 5 February and she was admitted to the hospital on 7 February. The case is of interest because it points out at least two issues related to diagnostic testing. First, the SARS-CoV-2 RT-PCR is an imperfect test that depends on the type, quality and handling of the sample, duration and severity of illness and assay performance. In general, lower respiratory tract samples have higher yields than upper respiratory tract ones [4]. Second, the low viral burden seen in some patients in the upper respiratory tract can make virologic diagnoses difficult, so that repeated sampling of multiple sites, testing of lower respiratory tract secretions, and, later in the course, detection of IgM/IgG antibodies may be necessary. Another point raised by the authors is the importance of the epidemiological link, in other words, the likely exposure within her family, in leading to prompt intervention, along with clinical features and radiological findings. The patient was admitted quickly and started on two putative antivirals on the same day. Whatever the chosen strategy, timely antiviral and supportive therapy is a major element for improving outcomes in SARS-CoV-2 illness. In parallel, some biomarkers, as illustrated in this patient, have demonstrated their value in evaluating the clinical course of this infection: lymphocyte count, CD4 and CD8 population subsets, LDH and CRP. Monitoring these laboratory measures, together with plasma D-dimer and IL-6 levels that have implications for treatment and prognosis, can prove to be of help in clinical management.

Li *et al.* reported, both in a short communication [5] and in a special report [6], some concern on the application of genomic evolutionary theories to SARS-CoV-2. The new coronavirus SARS-CoV-2, isolated from humans for the first time in December 2019, appears to have originated from bats. This was based on genetic analyses and comparisons with the sequences of other coronaviruses from different animal species. Specifically, two bat coronaviruses share 88% of the genetic sequence with that of SARS-CoV-2. In comparison, SARS-CoV-2 shares approximately 79% genetic sequence homology with SARS-CoV and 50% with MERS-CoV. As with SARS-CoV and MERS-CoV, it is assumed that the transmission did not occur directly from bats to humans, but that there could be another animal yet to be identified that acted as an intermediate host in transmission of the virus to humans. From a molecular point of view, the fact that coronaviruses can infect different animal species and humans can depend on at least two factors:

- Mutations that lead to substitutions in the virus surface protein (the spikes or ‘spikes’ of the virus) that favors the attachment of the virus to the cell receptors of the new host and its entry into the cell to replicate.
- Possibility of entry into the cell independent of the link between the viral protein and receptor as an alternative route for transmission between the different animal species and humans.

The point raised by Li *et al.* is that the application of evolutionary theories to ssRNA viruses – such as SARS-CoV-2 – could overestimate the divergence between a virus isolated in animal species and humans. SARS-CoV-2 is a positive-strand RNA virus that reproduces its genomic RNA through the action of an RNA dependent RNA polymerase and does not have an intermediate DNA genome like HIV. Moreover, RNA viruses adapt to the host expression system, in other words, their codons adapt to the host system they are infecting. In detail, the authors point out that SARS-CoV-2 and RaTG13, a bat coronavirus, were previously reported to have 17% synonymous changes at neutral sites [7]. Through the alignment of coding sequences of both viruses, Li *et al.* report that the RNA modification system in host cells might be the cause of 87% of the synonymous substitutions between SARS-CoV-2 and RaTG13 and conclude that an overestimation of virus divergence is a risk when comparing two RNA viruses [5].

Li *et al.* used several molecular techniques to investigate protein structures of SARS-CoV-2 [8]. The virus itself possesses five keys proteins: open-reading frame Lab (orfLab) in the nonstructural region and other four structural proteins, namely S (spike), M (membrane), E (envelope) and N (nucleoprotein) proteins. The S protein is part of the shell component and, as indicated above, a pivotal component for cell attachment. This protein is divided into two subunits, S1 and S2: S1 contains the receptor-binding domain (RBD) that binds to the angiotensin-converting enzyme 2 (ACE2) cellular receptor and S2 mediates virus-cell membrane fusion. The authors obtained the complete sequences of SARS-CoV-2 and six other bat coronaviruses. They applied multiple techniques including the analysis of structure and, most interestingly, the back mutation of some codon substitutions within the RBD. All SARS-CoV-2 five key proteins have a large homology with SARS-CoV from bats, although the lowest homology was with the S protein, which showed the highest amino acid homology with bat SARS RaTG13 reaching 97.71%. The authors concluded that it is very likely that SARS-CoV-2 developed from bat SARS CoV given this high similarity. Moreover, the authors backmutated the changed three amino acid residues (Glu^470^, Gln^484^ and Asn^487^) within the RBD structure and demonstrated that the mutated RBD structure has a stronger effect on ACE2 binding. This report adds to the considerable data on the crystal structures and biophysical characterization of the ACE2 interaction with the SARS-CoV-2 RBD that have been reported previously, including receptor conformational dynamics and other analyses highlighting similarities with the viruses from bats.

Hu *et al.* [9] have used a molecular docking software program to screen 109 plant-derived chemical agents for their ability to dock with the SARS-CoV-2 main protease (Mpro). As they point out in this Research Article, the recent characterization of the crystal structure of Mpro and its high degree of conservation across coronaviruses makes it an attractive target for drug discovery. They determined that the flavonoid rutin fits well into the Mpro substrate-binding pocket and, in addition, interacts with pockets in the toll-like receptors (TLRs), TLR2, TLR6 and TLR7. The finding that remdesivir, which targets the viral RNA-dependent RNA polymerases of coronaviruses and several other RNA virus families, also proved to be positive in the Mpro docking model raises some questions about its specificity. The nature of the interactions with the TLRs, whether stimulatory or inhibitory of proinflammatory responses and antiviral effects, are unclear from the report. The authors went on to assess host target genes of rutin by another modeling system and found multiple hits involving cellular functions, some of which are involved with inflammation. The authors speculate that rutin might have the potential to provide both antiviral and anti-inflammatory effects. While it is clear that this phytochemical has multiple pharmacological activities, as reviewed previously [10], this *in silico* report does not provide biologic data on rutin’s possible effects in SARS-CoV-2 infection.

Tan *et al.* discuss the critical public health issue of the extent to which asymptomatically infected persons are transmitting SARS-CoV-2 to others and contrast the currently available data with the epidemiologic findings in SARS and MERS [11]. Detection of relatively high SARS-CoV-2 RNA loads in upper respiratory tract samples has been reported in both presymptomatic (late incubation period) and truly asymptomatic infected persons. The proportion of asymptomatic infections has ranged widely from approximately 5–60% across various reports, but some studies have not had sufficient follow-up to document the development of later onset of symptoms. The factors that might impact on likelihood of asymptomatic infection (e.g., upper vs lower respiratory tract virus exposure, infectious virus inoculum size, pre-existing, cross-reactive immunity) have not been clarified. Of note, a large portion of pandemic and seasonal influenza infections are asymptomatic or very mild and detected retrospectively in serologic studies. The extent to which such persons contribute to transmission remains uncertain. However, studies of COVID-19 clusters have implicated the importance of pre- or asymptomatic persons in transmission, so that control measures clearly need to expand testing and isolation beyond those with symptoms. A recent modeling study estimated that 44% (95% CI: 25–69%) of secondary cases were infected during the presymptomatic stage of index cases [12]. Such findings, again, highlight the importance of wide-scale, rapid detection of SARS-CoV-2 infections for effective application of traditional nonpharmaceutical control measures (e.g., masking, social distancing, quarantine of those possibly exposed).

Following a broad-ranging introduction, Nemunaitis *et al.* focus on the strategy of repurposing a personalized cancer treatment with the proprietary name Vigil^®^ currently in Phase III trials to treat SARS-CoV-2 infections [13]. The cancer therapy involves harvesting tumor cells from a patient, transfecting with a dual shRNA construct that both inhibit furin production and stimulate GM-CSF expression to enhance immunogenicity of the tumor cells, and reintroducing them into the patient. The authors point out that the dual actions of their plasmid could result in both antiviral and anti-inflammatory effects in COVID-19. However, the cancer treatment strategy is a very different proposition from *in vivo* transfection of lung epithelial cells by their proposed inhalation delivery of the plasmid. Effective and safe aerosol delivery of small-molecular weight antivirals has proven very challenging in viral pneumonias to date, and the fragile nature of DNA plasmids adds complexity to the process. Depending on timing, there is also the concern that nonspecific GM-CSF stimulation in lung might exacerbate ARDS. Indeed, there is great interest in the use of immunomodulators in the subgroup of COVID-19 patients who develop a hyperinflammatory state associated with lung injury and often other organ failure. A large number of clinical studies targeting excessively proinflammatory immune responses are currently in progress in COVID-19. One agent of particular interest is baricitinib, an oral inhibitor of JAK1 and JAK2 and NAK family members that is approved for the treatment of rheumatoid arthritis in adults. It has been proposed as a therapeutic option for COVID-19 because of its anti-inflammatory and anticoronaviral properties in preclinical and limited clinical studies [14]. An obvious strategy is to combine antivirals with immunomodulators in severe COVID-19, and a randomized, placebo-controlled trial of baricitinib’s efficacy in combination with remdesivir is ongoing at present.

In summary, this special issue addresses many different aspects of the ongoing pandemic. Given the numerous unanswered questions regarding SARS-CoV-2 and COVID-19, we trust that this collection of articles will stimulate both discussion and further investigation. We thank the authors, reviewers and the staff at *Future Virology* for making this special issue possible.
